# Perspectives From Authors and Editors in the Biomedical Disciplines on Predatory Journals: Survey Study

**DOI:** 10.2196/13769

**Published:** 2019-08-30

**Authors:** Andrew J Cohen, German Patino, Puneet Kamal, Medina Ndoye, Anas Tresh, Jorge Mena, Christi Butler, Samuel Washington, Benjamin N Breyer

**Affiliations:** 1 Department of Urology University of California-San Francisco San Francisco, CA United States

**Keywords:** predatory journals, open access publication, global, citation, literature

## Abstract

**Background:**

Predatory journals fail to fulfill the tenets of biomedical publication: peer review, circulation, and access in perpetuity. Despite increasing attention in the lay and scientific press, no studies have directly assessed the perceptions of the authors or editors involved.

**Objective:**

Our objective was to understand the motivation of authors in sending their work to potentially predatory journals. Moreover, we aimed to understand the perspective of journal editors at journals cited as potentially predatory.

**Methods:**

Potential online predatory journals were randomly selected among 350 publishers and their 2204 biomedical journals. Author and editor email information was valid for 2227 total potential participants. A survey for authors and editors was created in an iterative fashion and distributed. Surveys assessed attitudes and knowledge about predatory publishing. Narrative comments were invited.

**Results:**

A total of 249 complete survey responses were analyzed. A total of 40% of editors (17/43) surveyed were not aware that they were listed as an editor for the particular journal in question. A total of 21.8% of authors (45/206) confirmed a lack of peer review. Whereas 77% (33/43) of all surveyed editors were at least somewhat familiar with predatory journals, only 33.0% of authors (68/206) were somewhat familiar with them (*P*<.001). Only 26.2% of authors (54/206) were aware of Beall’s list of predatory journals versus 49% (21/43) of editors (*P*<.001). A total of 30.1% of authors (62/206) believed their publication was published in a predatory journal. After defining predatory publishing, 87.9% of authors (181/206) surveyed would not publish in the same journal in the future.

**Conclusions:**

Authors publishing in suspected predatory journals are alarmingly uninformed in terms of predatory journal quality and practices. Editors’ increased familiarity with predatory publishing did little to prevent their unwitting listing as editors. Some suspected predatory journals did provide services akin to open access publication. Education, research mentorship, and a realignment of research incentives may decrease the impact of predatory publishing.

## Introduction

Increased access to the Internet has allowed for open access publishing to flourish. Traditional modes of scholarly publication involve the transfer of copyright from authors to publishers, with journal fees collected to provide access to articles. Traditional publishing, whether due to cost or perceived prejudicial peer review, is not embraced by all [1,2]. In contrast, in an open access model, authors typically retain rights to their work, it is immediately available to all readers, and funding is provided by authors themselves in the form of publication fees. Almost exclusively, publication and circulation of open access articles occurs through the Internet. Both models involve peer review, editing, and some degree of article promotion.

So called “predatory” journals take advantage of the open access publication model to prey on unsuspecting authors [3]. Such journals advertise the same services as open access journals but may fail to provide adequate peer review, licensing, quality control, and content preservation. Predatory journals are more likely to solicit authors via email for papers in exchange for publishing fees. Given that monetary incentives comes from author submissions, they are not beholden to the same motivation of high, perceived journal quality to entice library subscription as in traditional journals. By design, early-career physicians may not suspect such journals as illegitimate and are strongly incentivized to publish to advance their careers [4]. Predatory journals’ articles are not typically indexed on accepted forums and are objectively poor venues for dissemination. Predatory journals go to great lengths to appear legitimate; the fact that 400,000 items are published online per year under their banner speaks to their success [5].

Defining what is and is not a predatory journal has been a point of contention among researchers [6-8]. Beall’s list, a list of publishers thought to be possibly predatory based on a single researcher’s criteria, has been controversial [8]. It is now only available as an online resource. Recently, other groups have independently identified characteristics that suggest a journal is predatory [9]. Lists that purposefully provide certification of legitimate open access publishing, such as the Directory of Open Access Journals (DOAJ) [10] and the Open Access Scholarly Publishers Association (OASPA) [11], offer an alternative way to ensure high-quality open access publishing. Up-and-coming open access journals could appear to share some of the characteristics of a predatory journal and have been critical of blacklists [7,12].

What remains unknown amid this controversy is the perspective of both editors who manage predatory journals and those choosing to publish in them. While the lay press and literature has increasingly drawn attention to this issue, these journals continue to exist [3,5,13]. No literature to date addresses whether authors are unknowing victims or complacent coconspirators in the predatory publishing scheme. Our objective was to understand the motivation of authors in sending their work to potentially predatory journals. Moreover, we aimed to understand the perspective of journal editors at journals publicly cited as potentially predatory. We hypothesize that authors and editors are unaware that journals in which they are involved are possibly predatory.

## Methods

Beall’s list [8] was accessed on August 1, 2018, at which time 2567 publishers were noted. Beall’s list was created using 48 criteria for a publisher-at-large or an individual journal [8]. The criteria assess varied factors such as grammatical or spelling errors on official communications, availability of complete editorial contact information, and the presence of false claims of indexing by services such as PubMed [14]. At present, this list is exclusively available online [8].

**Figure 1:**
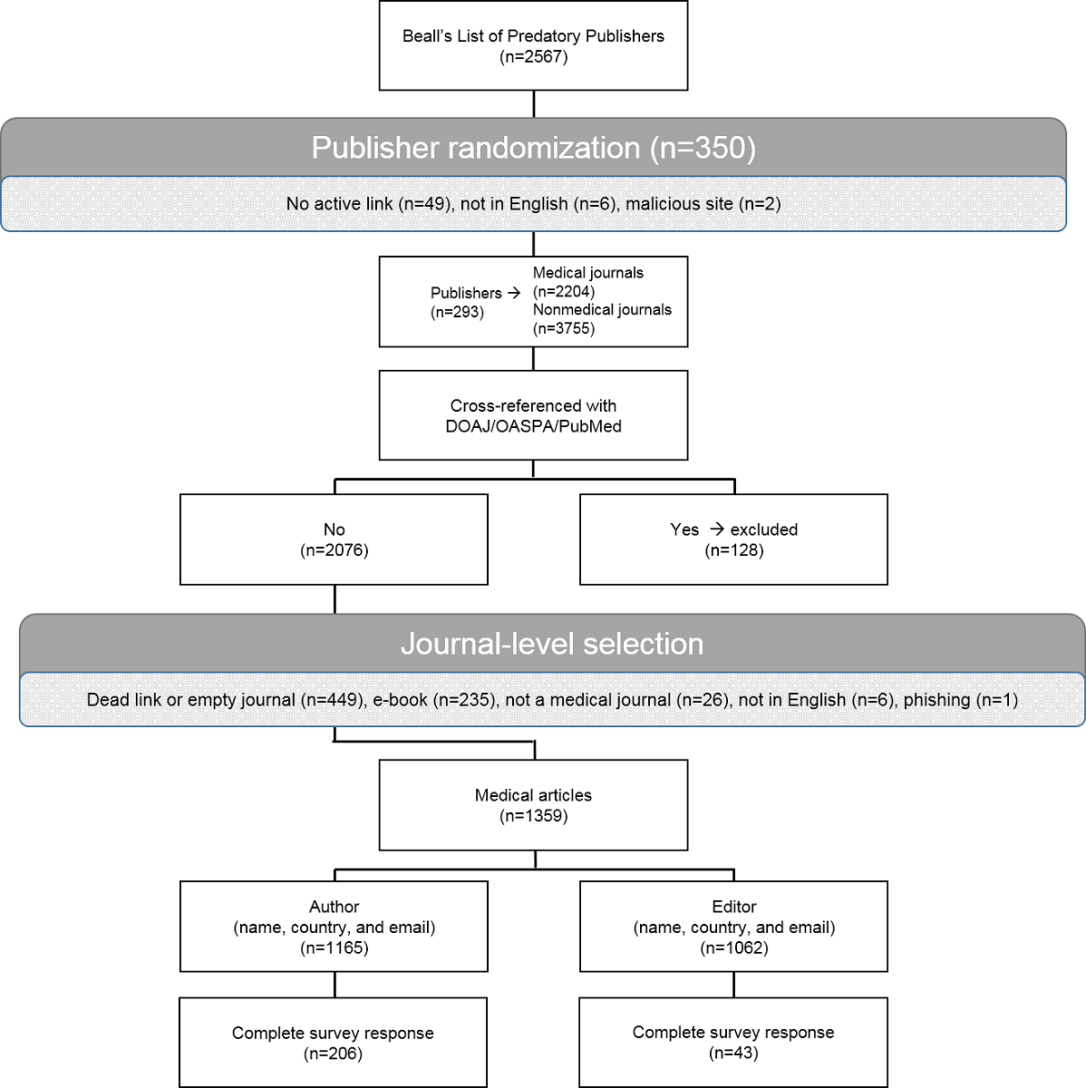
The CONsolidated Standards Of Reporting Trials (CONSORT) flow diagram. DOAJ: Directory of Open Access Journals; OASPA: Open Access Scholarly Publishers Association.

A total of 350 publishers were randomly selected from this list, using a random-number generator. Eight reviewers (AC, GP, PK, MN, AT, JM, CB, and SW) evaluated publishers using the following criteria. First, we confirmed an active link to the publisher website. Second, we reviewed the titles of journals within the portfolio of a publisher’s work to assess if any were biomedical journals in scope. Specifically, we applied the MEDLINE criteria: “[Journals] predominantly devoted to reporting original investigations in the biomedical and health sciences, including research in the basic sciences; clinical trials of therapeutic agents; effectiveness of diagnostic or therapeutic techniques; or studies relating to the behavioral, epidemiological, or educational aspects of medicine” [15]. Journals meeting this criteria were noted. Third, we cross-referenced listed journals with the DOAJ [10], the OASPA [11], and the US National Library of Medicine [14]. We excluded journals listed on any of these sites. Fourth, we assessed whether links to specific journals were live and whether the journals had published articles. Fifth, the reviewers selected the most recent, original online article from the journal and recorded the name, country of origin, and email of both author and editor from the website. The selection criteria and ultimate composition of the cohort are summarized in Figure 1. All data were stored in REDCap [16].

Surveys for both authors and editors were created during a round-table discussion among study authors. The surveys underwent iterative testing for clarity, content, and length. Next, we invited editors and authors to participate via email. Surveys were administered using REDCap, with one reminder sent to nonresponders. We simultaneously performed a nested randomized controlled trial on incentives to encourage survey response, which is published separately [17]. Invitees were randomized into a control group, a group eligible for a cash prize (US $100), and a group whose response lead to monetary donation to charity (US $2.50 to Rotary International per respondent). Rotary International is a nondenominational international charity with 35,000 worldwide clubs that have been instrumental in multiple projects, including the fight to eradicate Polio [18]. To maximize recruitment, survey invitations were personalized to include the article and journal name for authors and the journal name for editors.

Surveys assessed authors’ basic demographics, publication history, their recollection of the editorial process for the article in question, and their knowledge regarding predatory journals (see Multimedia Appendix 1). Editors were queried about basic demographics, the editorial process for the publication in the journal in question, cost of publication, and their knowledge regarding predatory journals (see Multimedia Appendix 2). Of note, the mention of predatory journals was not made until a separate page of the survey, so as to not prejudice responses. For the purposes of this study, we defined a predatory journal as “an exploitative open-access academic publishing business model that involves charging publication fees to authors without providing the editorial and publishing services typically associated with legitimate journals.” Citations of articles as reported by authors were confirmed using a search in Google Scholar [19]. Partial survey responses were excluded, but those answering *Prefer not to answer* were not considered incomplete responses.

Summary statistics were used to describe the cohort. Means and standard deviations as well medians and interquartile ranges (IQRs) were used for continuous variables. Frequency tables were used for categorical variables. The chi-square statistic was used to compare frequencies between groups. Developed-nation status was based on the World Bank listing for high-income countries derived from gross national income higher than US $12,056 per capita [20]. Statistics were calculated using Stata 15 (StataCorp). The datasets used and/or analyzed during this study are available from the corresponding author upon reasonable request.

Consent for publication was granted by participants when they responded to the survey in accordance with Institutional Review Board (IRB) approval. Ethical clearance was granted by the IRB of the University of California, San Francisco, CA (approval number: 18-25351).

## Results

### Overview

**Figure 2:**
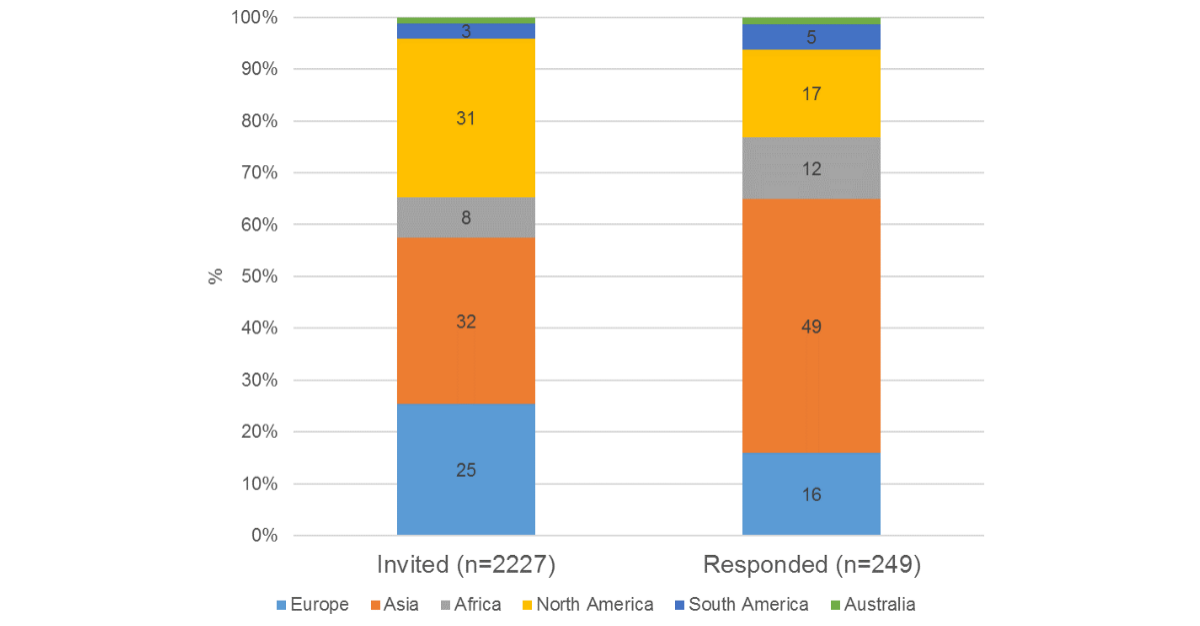
Geographic distribution of survey invitees and responses.

Journals meeting selection criteria came from 181 distinct publishers. There was a substantial range in the number of journals per publisher represented in our cohort. A total of 58.0% of publishers (105/181) were singular entities, having only a single journal in their portfolio. However, several larger publishers were also included; indeed, 52.00% of (1146/2204) journals came from just 3 out of 181 publishing companies (1.7%). Of the articles selected, 59.68% (811/1359) were published in 2018, 17.73% (241/1359) were published in 2017, and none were published before 2014. Authors and editors represented a global academic community (see Figure 2). Of all articles selected, 40.03% (544/1359) had corresponding authors from high-income countries, as defined by the World Bank [20]. The overall survey response rate was 13.0%, with editor response rates significantly lower than those for authors (6.5% vs 18.9%, respectively, *P*<.01). Several respondents provided incomplete responses (n=40). Complete responses were collated from 206 authors and 43 editors, out of a potential 1165 and 1062, respectively, for a final response rate of 11.18% (249/2227).

### Authors

Responding authors had a median age of 43 years (IQR 33-54), with 47.0% (95/202) reporting that they had been in practice for over 15 years (see Table 1). A total of 7.4% (15/202) of authors self-identified as still in a training program. Among authors, 80.1% (165/206) reported that publication of articles is part of their academic promotion process. Authors published a median of 3 (IQR 2-6) manuscripts in the year prior to survey completion; lifetime median articles published was 15 (IQR 5-45). A total of 56.1% (110/196) of funding for research projects was from personal funds. Authors reported a median cost of US $190 (IQR 0-520) for publication. A total of 78.2% of authors (158/202) recalled a peer review, with 38.6% (76/197) reporting 16-30 days between submission and acceptance. A total of 68.0% of authors (140/206) had to submit revisions and 67.0% of authors (138/206) did not submit their article elsewhere before selecting this particular journal. Of 206 studies, 22.3% (n=46) were observational, 11.2% (n=23) were basic science studies, 10.7% (n=22) were case series, and 9.7% (n=20) were systematic reviews.

A total of 12.1% of authors (25/206) felt publication in their chosen journal was both prestigious and had a positive impact on their career. In addition, 19.6% of authors (39/199) noted a positive career impact if they reported their paper was cited versus 10.6% (21/199) that reported a positive impact when their paper was not cited (*P*=.04), with 27.2% (56/206) of total articles reportedly cited. Authors’ perceptions of the journals’ prestige was not impacted by whether their paper was cited. Google Scholar searching confirmed that only 16% (9/56) of those articles purported to be cited had a recorded citation. A total of 40.8% of authors (84/206) felt this particular publication was neutral in terms of career advancement and 14.1% (29/206) had published in the same journal prior to this particular publication. Most common reasons for selecting this particular journal for publication were open access (74/206, 35.9%), solicitation (52/206, 25.2%), and affordability (31/206, 15.0%).

Authors that paid fees in the top quartile (>US $519) for publication had no difference in perception of journal prestige, impact on career, shorter publication times, or fewer revision requests than authors paying lower fees (see Table 2). They were significantly more likely to use private, nongovernment funds (*P*<.01). These authors reporting higher expenses were not necessarily more likely from high-income countries (80/206, 38.8% vs 60/206, 29.1%; *P*=.08). Authors came from 54 countries, spanning all continents except Antarctica; 23.3% (48/206) were from India and 13.6% (28/206) were from the United States.

### Editors

Editors’ median age was 41 years (IQR 33-53) (see Table 1). Among survey responders, 40% of editors (16/40) were from India, 20% (8/40) were from the United States, and the residual editors were from varied locations. Editors reported performing editorial duties for a median of 2 journals (IQR 2-4), and for 79% of the editors (34/43), such involvement promotes their academic advancement. A total of 42% of editors (18/43) work primarily in an academic center and 79% (34/43) noted that being an editor positively impacts their career.

**Table 1:** Authors’ and editors’ basic demographics.

Demographics		Authors (N=206)	Editors (N=43)	*P* value
Age in years, median (IQR^a^)		43 (33-54)	41 (33-53)	.72
Gender (male; N=205 authors), n (%)		147 (71.7)	29 (67)	.43
From high-income country, n (%)		71 (34.5)	13 (42)	.40
**Years in practice (N=202 authors; N=42 editors), n (%)**				
	1-5	37 (18.3)	8 (19)	.77
	6-10	34 (16.8)	9 (21)	
	11-15	21 (10.4)	4 (9)	
	>15	95 (47.0)	20 (48)	
	In training	15 (7.4)	1 (2)	
Estimated publication cost in US $, median (IQR)		190 (0-520)	634 (75-1360)	.02
**Estimated days from submission to acceptance (N=197 authors; N=25 editors), n (%)**				
	0-15	30 (15.2)	4 (16)	.01
	16-30	76 (38.6)	4 (16)	
	31-45	45 (22.8)	14 (56)	
	46-60	25 (12.7)	2 (8)	
	>60	21 (10.7)	1 (4)	
Articles did not undergo peer review (N=25 editors), n (%)		45 (21.8)	2 (8)	N/A^b^
**Type of study (author), n (%)**				
	Observational	46 (22.3)	N/A	N/A
	Other^c^	32 (15.5)	N/A	
	Basic science	23 (11.2)	N/A	
	Case series	22 (10.7)	N/A	
	Survey research	20 (9.7)	N/A	
	Systematic review	20 (9.7)	N/A	
	Qualitative research	15 (7.3)	N/A	
	Cross-sectional	15 (7.3)	N/A	
	Editorial or letter to the editor	13 (6.3)	N/A	
**Reasons for publishing in this particular journal (author), n (%)**				
	Open access for dissemination	74 (35.9)	N/A	N/A
	Other^d^	52 (25.2)	N/A	
	Solicited by editor	52 (25.2)	N/A	
	Affordability	31 (15.0)	N/A	
	Influenced by online advertising	22 (10.7)	N/A	
	Recommendation from peer	22 (10.7)	N/A	

^a^IQR: interquartile range.

^b^Not applicable.

^c^Other types of studies include meta-analyses, randomized controlled trials, case controls, and critical reviews.

^d^Other reasons include impact factors, recommendations from supervisor, print ads, and perceived journal prestige.

**Table 2:** Publication costs and authors’ perceptions.

Perceptions and other factors		Bottom 75th percentile of cost	Top 25th percentile of cost	*P* value
**Perception of journal prestige (N=126 bottom 75th; N=71 top 75th), n (%)**				
	Not prestigious	19 (15.1)	9 (13)	.81
	Little prestige	35 (27.8)	15 (21)	
	Moderate prestige	52 (41.3)	34 (48)	
	Very prestigious	15 (11.9)	10 (14)	
	Most prestigious	5 (4.0)	3 (4)	
**Impact on career (N=129 bottom 75th; N=72 top 75th), n (%)**				
	Large negative impact	2 (1.6)	1 (1)	.49
	Small negative impact	3 (2.3)	1 (1)	
	Neutral	54 (41.9)	28 (39)	
	Small positive impact	51 (39.5)	24 (33)	
	Large positive impact	19 (14.7)	18 (25)	
**Estimated days from submission to acceptance (N=127 bottom 75th; N=70 top 75th), n (%)**				
	0-15	16 (12.6)	14 (20)	.69
	16-30	50 (39.7)	26 (37)	
	31-45	31 (24.4)	14 (20)	
	46-60	17 (13.4)	8 (11)	
	>60	13 (10.2)	8 (11)	
Revisions required (N=127 bottom 75th; N=72 top 75th), n (%)		85 (66.9)	51 (71)	.57
**Funding source (N=130 bottom 75th; N=66 top 75th), n (%)**				
	Personal	82 (63.1)	27 (41)	<.001
	Department	21 (16.2)	13 (20)	
	Private	5 (3.9)	15 (23)	
	Public	22 (16.9)	11 (17)	
Article subsequently cited (N=129 bottom 75th; N=71 top 75th), n (%)		33 (25.6)	21 (30)	.43

### Editors’ and Authors’ Views on Predatory Journals

A total of 40% of editors (17/43) who responded were not aware that they were listed as an editor for the particular journal in question. These editors were excluded from subsequent analysis. Among remaining editors, 96% (25/26) felt their journal was not a predatory journal and 50% (13/26) were already aware their journal was listed on Beall’s list. A total of 92% (24/26) of editors reported that their journal requires peer review. Editors reported that the mean cost to publish in their journals was US $634 (IQR 75-1360). Editors’ estimated, on average, that 50% of manuscripts required revisions and that 35% were rejected. A total of 54% (14/26) of editors reported that 31-45 days elapsed between submission and acceptance.

**Figure 3:**
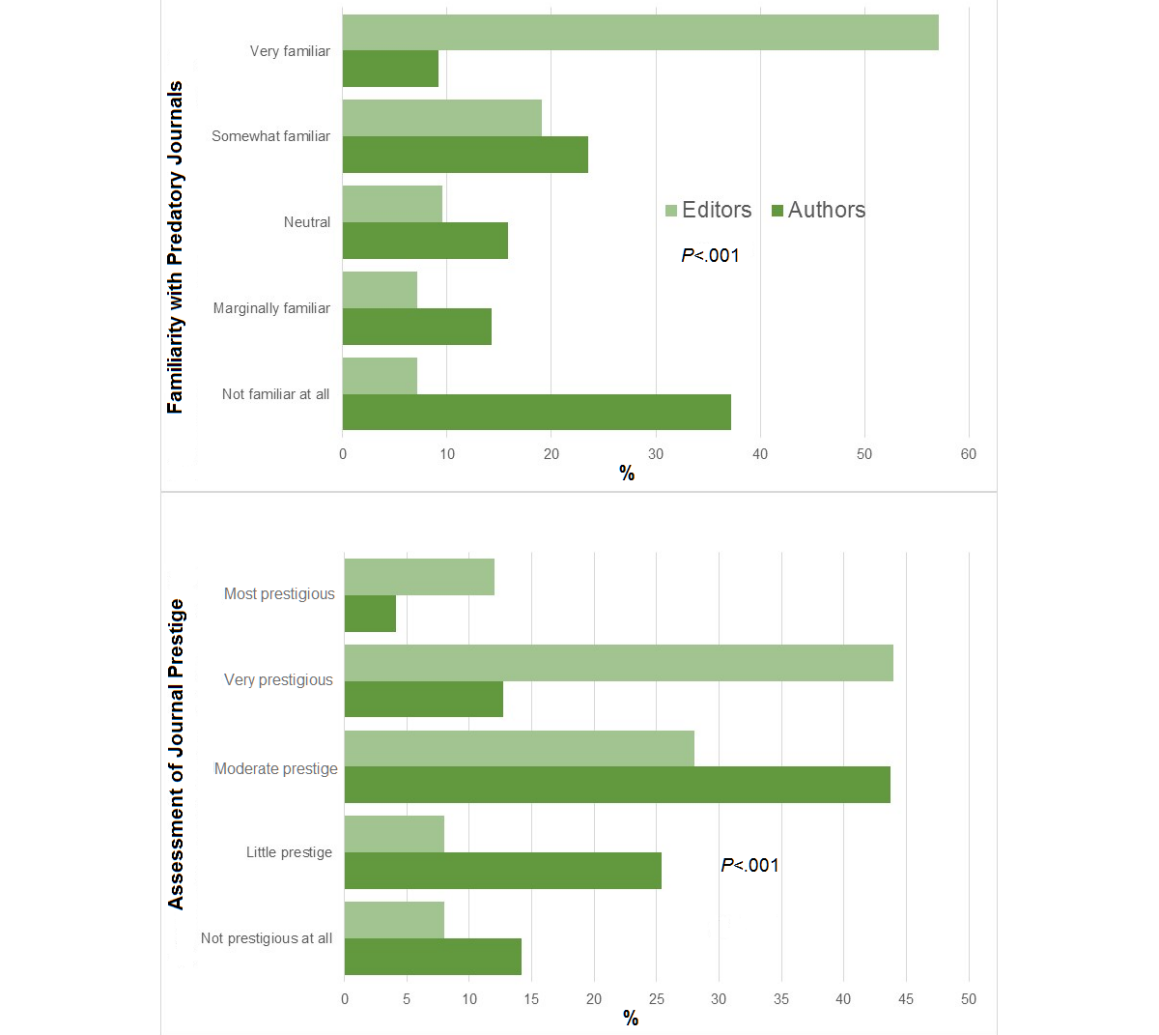
Discrepancy in author and editor opinions on predatory journals.

Whereas 77% of all surveyed editors (33/43) were at least somewhat familiar with predatory journals, only 33.0% (68/206) of authors were somewhat familiar with them (*P*<.001) (see Figure 3). Similarly, only 26.2% of authors (54/206) were aware of Beall’s list of predatory journals versus 49% (21/43) of editors (*P*<.001). A total of 30.1% of authors (62/206) believed that their publication was published in a journal that could be defined as predatory, once given the definition. Given knowledge that a journal was definitely predatory, 87.9% of authors (181/206) surveyed would not publish in the same journal in the future.

Narrative comments were also collected via the survey. These provided surprising and conflicting insight into the attitudes and opinions of both authors and editors. Additionally, many survey respondents felt passionate enough about this topic to contact the research staff via email to share additional long-form opinions. Selected comments are presented in an anonymized fashion in Multimedia Appendix 3. Some editors expressed surprise and outrage about their listing as editors, whereas others were defensive of their journals, described continued editorial improvements, and in rare cases disparaged Beall’s list. Authors expressed regret about their publication choice, whereas others felt they had no other options or provided negative commentary about traditional publishing models. The comments emphasize the controversial and divergent opinions surrounding predatory publishing. These issues will not be solved overnight; as stated by one author, “In [country name], one must have two or three publications in [a] journal with impact, so you do not say what or why; simply you must do it.”

## Discussion

### Principal Findings

We found editors were at least somewhat familiar with predatory journals, but the vast majority of authors (67%) were not. Per authors and editors, in limited cases, predatory journals seemingly provide some editorial support via revisions, rejections, and circulation that was enough to drive citations. Predatory journal authorship is a global phenomenon in our study, with higher penetrance in India and the United States. Alarmingly, 39% of editors reported not even being aware of being a journal editor for the journal in question. Several editors sent us comments stating they had previously asked that their name and contact information be removed from the journal websites, given no purposeful affiliation. After alerting authors that their recent publication was in a potentially predatory journal, 88% would avoid the same journal, demonstrating that via dissemination of knowledge regarding predatory practices, predatory publishing may decrease.

### Services Provided by Predatory Journals

On the surface, these predatory journals are providing at least some service to authors. In our cohort, authors frequently recalled a peer review and a need to submit revisions. Editors similarly stated that a peer review was performed and approximately 35% of articles were rejected. A potential marker of journal prestige and circulation is ultimately citation, reported by 27% of surveyed authors [21]. Of note, we could only verify 16% of author-reported citations. While assessing the quality of dissemination or peer review is beyond the scope of this work, from provided comments, there is clearly a stigma with being associated with a predatory journal for both authors and editors. There is little debate that any journal continuing to list editors that renounce that affiliation are illegitimate. Nevertheless, this work urges caution in branding a journal predatory without insight provided by editors and authors; ongoing work defining predatory journals should incorporate their perspectives.

### Further Analysis

We found that 40% of authors were from countries designated by the World Bank as high income; prior work suggests that authors who published in predatory journals were primarily from developing nations [22]. A predominance of Indian (34.7%), African (16.4%), and Asian (25.6%) authorship was shown previously, using a complex stratified selection criteria of publishers; in our cohort, 23% of authors were from India [5]. Recent growth in research enterprise in nations such as India has created a need for forums of publication. Publication in the mainstream scientific press may be limited by access, bias against international research work, lack of shared scientific interest, or a true marker of low-quality work [12]. India may be a case study for this situation and this could explain why it is overrepresented in authors and editors in predatory journals, as typical journals do not and cannot accept the quantity of work produced [23].

Prior work proposed that young naïve authors are the target of predatory journals, but 47% of responding authors in this study have been in practice for more than 15 years [4]. Similarly, in economics, a surprising 11% of 1284 articles in predatory journals were published by authors registered as part of a prestigious international authorship group [24]. By way of strategy, publishers target emerging markets rather than new researchers specifically [25]. By creating multiple publishing sites, each with 60-100 unrelated journals tailored to a particular culture, it can be difficult to complete an online journal search and not link to one of these journals. Further analysis suggested that such journals essentially had the same editorial team, despite glaringly different topic areas and very few, if any, publications [25].

A high proportion of responding authors in our cohort reported that publication is an important facet of academic advancement. There is a growing concern that the motivation to publish may supersede desires for research quality [26]. Both authors and editors are incentivized to publish and edit to advance their careers. While the scholarly advancement of knowledge is a noble pursuit in and of itself, a growing number of scientific articles are never cited, suggesting inherently that they are flawed, do not contribute to knowledge, or are in poorly indexed journals rendering their research essentially invisible [27]. It is a fact that due to limited resources and human capital, not all academic centers are set up to succeed as a research enterprise. This may limit the potential impact of research from such sources. Mixed with incentives to “publish or perish,” authors may be tempted to lower their standards for publication and provide a market for continued predatory publishing.

Potential solutions to predatory publishing include demanding promotion committees judge faculty on the quality of publications in lieu of sheer number [27]. Authors should receive training regarding the existence of predatory publishers. The onus remains on authors to independently verify credentials of journals and to confirm journal indexing claims. New initiatives such as *Think. Check. Submit.* [28], an international collaboration providing practical resources to educate researchers, promote integrity, and build credible research publications, should be publicized [29]. Research mentors should assist colleagues and steer them toward legitimate journals. White lists or other affirming criteria should be publicized among authors seeking publication to motivate higher-quality outlets for their work. Authors in our study were most motivated by cost and the open access model, so high-quality journals meeting that criteria should be lauded. They were also motivated by solicitations, so education surrounding such email or print invitations should be circulated [30]. Accountability for publishers should be demanded, particularly for blatant false claims involving indexing, editors, and cost. Regulatory oversight in the country of origin of predatory publishers should be strengthened—in some cases, no such oversight exists—and criminal charges pursued, if relevant.

Some authors shared already-published concerns regarding blacklists, such as Beall’s stifling of innovation in open access publishing, which is why we cross-referenced our findings with well-publicized white lists to generate a cohort of authors and editors [6-8]. Prior work on predatory journals has not made this effort [5-8]. White lists provide an independently verified listing of journals with ethical and quality publication standards. We elected to use the US National Library of Medicine [14] as one white-list source in lieu of Embase or other publisher-operated sites. Given that Embase and similar sites are publishing-company owned, their ability to assess the suitability of journals for inclusion may be biased, given their own financial incentives. In contrast, the US National Library of Medicine has transparent requirements for inclusion. Nonetheless, different white lists could provide a slightly different cohort of authors or editors to study and, hence, caution is advised in drawing generalizations.

### Limitations

A major limitation to this study is our poor response rates from both authors and editors, but particularly editors. Given that 39% of responding editors were not even aware that they were editors at the journal in question, it is not surprising that editors-at-large may have faced confusion with our survey invitation. Moreover, unlike author email addresses, the editorial email addresses often took a generic form, such as “editor@journalname.org,” and may have encountered staff that did not forward the survey to the intended recipient. Our data comes from an international cohort, but we randomized the selection of publishers and not the countries of publishers. As such, some countries only contributed via a single survey response and the responses should not be seen as representative of all authors of that region. All authors published their papers in English journals. Nonetheless, English may not be a first language for respondents, limiting responsiveness or leading to response error. Due to monetary and time constraints, we did not perform survey adaptation or cross-cultural validation for global participants [31,32]. Survey response may have been low due to survey fatigue, disinterest, or prejudice against the topic area, which may have increased response bias [31,32]. Ultimately, this was a volunteer sample, which selected for respondents over nonrespondents. Given that our demographic data exclusively came from voluntary respondents, we cannot compare to the nonresponder group, reducing the generalizability of our work.

### Conclusions

Predatory journal authorship is a global phenomenon not unique to early-career researchers. The majority of studied authors were not familiar with predatory publishing practices, despite being published in a suspected predatory journal. Alarmingly, 39% of editors reported that they were not even aware of being an editor for the journal in question, clearly confirming the unethical practices of such journals. Education, research mentorship, and a realignment of research incentives may decrease the impact of predatory publishing.

## Appendix

Multimedia Appendix 1 Author online survey.

Multimedia Appendix 2 Editor online survey.

Multimedia Appendix 3 Free-form comments, edited for length, grammar, and spelling.

## Abbreviations

CONSORT: CONsolidated Standards Of Reporting Trials
DOAJ: Directory of Open Access Journals
IQR: interquartile range
IRB: Institutional Review Board
OASPA: Open Access Scholarly Publishers Association
