# Supplementary material for: Perspectives From Authors and Editors in the Biomedical Disciplines on Predatory Journals: Survey Study
Source: J Med Internet Res. 2019 Aug 30;21(8):e13769. doi: 10.2196/13769 (PMC6743260; doi:10.2196/13769)
Supplement: Multimedia Appendix 1 [file jmir_v21i8e13769_app1.pdf]

Questionnaire for Authors

Please complete the survey below.

Thank you!

---

1) What is your age?

____
(Please enter a number)

---

2) What is your gender?

◯ Male ◯ Female ◯ Other
◯ Prefer not to answer

---

3) How many years have you been in practice?

◯ In training
◯ 1-5
◯ 6-10
◯ 11-15
◯ >15

---

4) Is academic production or publication of articles necessary for academic promotion at your facility?

◯ Yes
◯ No

---

5) How many articles did you publish in peer reviewed journals last year?

____
(Please enter a number)

---

6) How many articles have you published in peer reviewed journals during your career?

____
(Please enter a number)

---

7) Who funded this study?

◯ Personal funds
◯ Department Funds
◯ Public (Government) Grant
◯ Private (Non-Government) Grant

---

8) About how much did it cost to publish the study in this journal?

____
(Please enter an estimate in US Dollars)

---

9) Did your article, "[article_title]" undergo peer review by the editorial staff of the [journal_title]?

◯ No
◯ Yes
◯ Unsure

---

10) About how many days elapsed between submission and acceptance?

◯ 0-15
◯ 15-30
◯ 31-45
◯ 46-60
◯ >60

---

11) Were you required to submit revisions?

◯ Yes
◯ No

12) Prior to acceptance at this journal, how many journals declined to accept this publication?

- ◯ 0
- ◯ 1
- ◯ 2
- ◯ 3
- ◯ 4
- ◯ 5 or more

13) How would you characterize the type of the published study?

- ◯ Observational
- ◯ Survey Research
- ◯ Qualitative Research
- ◯ Case Series
- ◯ Cross-Sectional
- ◯ Case-Control
- ◯ Randomized Control Trial
- ◯ Basic Science
- ◯ Meta-analysis
- ◯ Systematic Review
- ◯ Critical Review
- ◯ Editorial or Letter to the Editor

14) How would you rate the prestige of this journal?

- ◯ Not prestigious at all
- ◯ Little prestige
- ◯ Moderate Prestige
- ◯ Very Prestigious
- ◯ Most Prestigious

15) How has this publication impacted your career?

- ◯ Large Negative Impact
- ◯ Small Negative Impact
- ◯ Neutral Impact
- ◯ Small Positive Impact
- ◯ Large Positive Impact

16) Has this article been cited by others?

- ◯ No
- ◯ Yes
- ◯ Unknown

17) Have you published in this journal before?

- ◯ No
- ◯ Yes

18) Why did you choose to publish in this particular journal ?

- ☐ Prestige
- ☐ Influenced by Online advertising
- ☐ Influenced by Print advertising
- ☐ Recommendation from peer
- ☐ Recommendation from superior
- ☐ Solicited by editor
- ☐ Affordability
- ☐ Influenced by Impact Factor
- ☐ Open Access for dissemination

19) Are there any other reasons you published in this particular journal?

____

20) How familiar are you with 'predatory journals' ?

- ◯ Not familiar at all
- ◯ Marginally familiar
- ◯ Neutral
- ◯ Somewhat familiar
- ◯ Very familiar

A Predatory Journal is an exploitative open-access academic publishing business model that involves charging publication fees to authors without providing the editorial and publishing services typically associated with legitimate journals.

21) Based on your experience with this recent publication do you believe [journal_title] is a predatory journal?

◯ Yes
◯ No

22) Are you familiar with Beall's List of Predatory Journals?

◯ Yes
◯ No

23) If you knew this journal was definitely a predatory journal, how likely would you be to publish there again?

◯ Very Unlikely
◯ Unlikely
◯ Neutral
◯ Likely
◯ Very Likely

24) Please provide any other comments you may have on this topic.

____
