# Supplementary material for: Perspectives From Authors and Editors in the Biomedical Disciplines on Predatory Journals: Survey Study
Source: J Med Internet Res. 2019 Aug 30;21(8):e13769. doi: 10.2196/13769 (PMC6743260; doi:10.2196/13769)
Supplement: Multimedia Appendix 2 [file jmir_v21i8e13769_app2.pdf]

Questionnaire for Editors

Please complete the survey below.

Thank you!

What is your age?

____
(Please enter a number)

What is your gender?

- ◯ Male ◯ Female ◯ Other
- ◯ Prefer not to answer

How many years have you been in practice?

- ◯ In training
- ◯ 1-5
- ◯ 6-10
- ◯ 11-15
- ◯ >15

In what setting is your primary practice ?

- ◯ Academic Hospital
- ◯ Community Hospital
- ◯ National Level Hospital
- ◯ Private Hospital
- ◯ Non-Clinical or Research Only Duties
- ◯ Ambulatory Care Only

Were you aware that you are listed as an Editor for [journal_title]?

- ◯ Yes
- ◯ No

Does being a journal editor impact your academic promotion?

- ◯ Yes
- ◯ No

Please estimate the number of journals for which you are listed as an editor.

____
(Please enter a number)

About how much does it cost to publish a study in this journal?

____
(Please enter an estimate in US Dollars)

Do all articles undergo peer review by the editorial staff of the journal?

- ◯ No
- ◯ Yes
- ◯ Unsure

About how many days elapse between submission and acceptance?

- ◯ 0-15
- ◯ 15-30
- ◯ 31-45
- ◯ 46-60
- ◯ >60
- ◯ Unsure

About what percentage of potential manuscripts are rejected?

____
(%)

About what percentage of potential manuscripts require revisions?	__________________________________ (%)
Considering all of your work related tasks, what percentage of your time is directly related to editorial duties?	__________________________________ (%)
How would you characterize the majority of published work in the journal over the past year?	◯ Observational<br>◯ Survey Research<br>◯ Qualitative Research<br>◯ Case Series<br>◯ Cross-Sectional<br>◯ Case-Control<br>◯ Randomized Control Trial<br>◯ Basic Science<br>◯ Meta-analysis<br>◯ Systematic Review<br>◯ Critical Review<br>◯ Editorial or Letter to the Editor
How would you rate the prestige of this journal?	◯ Not prestigious at all<br>◯ Little prestige<br>◯ Moderate Prestige<br>◯ Very Prestigious<br>◯ Most Prestigious
How has being an editor of this publication impacted your career?	◯ Large Negative Impact<br>◯ Small Negative Impact<br>◯ Neutral Impact<br>◯ Small Positive Impact<br>◯ Large Positive Impact
How familiar are you with 'predatory journals' ?	◯ Not familiar at all<br>◯ Marginally familiar<br>◯ Neutral<br>◯ Somewhat familiar<br>◯ Very familiar

A Predatory Journal is an exploitative open-access academic publishing business model that involves charging publication fees to authors without providing the editorial and publishing services typically associated with legitimate journals. Suspected predatory journals and publishers have been published in print and online by the controversial Beall's list.

In your opinion do you believe [journal_title] is a predatory journal?

- ◯ Yes
- ◯ No

A Predatory Journal is an exploitative open-access academic publishing business model that involves charging publication fees to authors without providing the editorial and publishing services typically associated with legitimate journals. Suspected predatory journals and publishers have been published in print and online by the controversial Beall's list.

Are you aware that this journal has been listed on Beall's List of Predatory journals?
- ◯ Yes
- ◯ No

Have you made any changes to the journal due to your knowledge of being listed as a potential Predatory Journal?
- ◯ Yes
- ◯ No

Please summarize any changes.

____

This journal, [journal_title], has been listed as a potential Predatory Journal. Would you plan to make any changes to the journal's publication process given knowledge of that listing?
- ◯ Yes
- ◯ No

Please summarize any changes.

____

Please provide any other comments you may have regarding this topic area.

____
