# Supplementary material for: Perspectives From Authors and Editors in the Biomedical Disciplines on Predatory Journals: Survey Study
Source: J Med Internet Res. 2019 Aug 30;21(8):e13769. doi: 10.2196/13769 (PMC6743260; doi:10.2196/13769)
Supplement: Multimedia Appendix 3 [file jmir_v21i8e13769_app3.pdf]

Appendix III- Free Form Comments (edited for length, grammar, and spelling)

In response from editors to "Have you made any changes to the journal due to your knowledge of being listed as a potential Predatory Journal?"

- 1.We are giving details of editorial board on website, 2.We are writing editorial regularly, 3.We are rejecting articles with plagiarism.
- Effective peer review process with active reviewers and editorial board.
- Increased review pattern and rejection percentage is increased. Using plagiarism software to check the quality of work
- All the articles underwent peer review and published after revision.
- Effective and efficient Editorial & Reviewer Board Members [with] rigorous peer review process

In response from editors to "Would you plan future changes to the journal's publication process given knowledge of that listing (on Beall's list)?"

- I will resign as editor
- By keeping good examiners, and removing the reviewers who accept the manuscripts without reviewing. We will try for potential manuscripts which help[] our journal impact [and] search for novel research
- Our journal is following [a] peer review process. We [have a] single blinded system; we need to make people aware of the review process.
- Go through a process which confirm genuine author and their work
- Ask to be removed as an editorial board member.
- This journal runs on a Not-for-Profit Model and funds collected are reinvested to improve journal services. We will charge a minimum publication fee to authors for providing the best editorial and publishing services.
- Follow standard guidelines of COPE, ICMJE and have a detailed plagiarism check.
- I will do my best to make it a top journal. I would have committee of eminent personalities along with the peer review group. All articles will be reviewed by peers of the concerned specialty and if found acceptable will be published. Nominal charges will be levied on the authors. [Desire] the Journal reaches every nook and corner of the world.

Any other comments (excluded trivial messages such as thanks):

- After acceptance the journal was unusually and extremely aggressive with asking for the payment.
- I indicated I would not pay any publication fees prior to agreeing to write. They accepted those terms
- By strict definition, [this journal] could be classified as predatory, and yet, having published in many traditional journals, I found the editorial process--and outcome--comparable. I didn't pay, and, it got strong material published and circulated that several traditional journals refused solely due to sphincter tone.
- The contents of this topic were very informative and I will be very much careful next time while submitting my valuable research. Thanks team for such efforts to enlighten us about dark sides of the research and academic activities
- There may be more journals for wide-spreading reviews. Nowadays, very few journals accept reviews and those accepting [have] little impact, thus this is a market for predatory journals
- I am very conscious of the existence of predatory journals; however, my superior felt the lists online are too 'all encompassing' and difficult to determine legitimacy. It would be beneficial to have a more rigorous list that all Universities provided to their staff.
- I am a retired scientist. My pension is small, since I was [an] immigrant. I pay for publications from my pocket or invite someone to pay for publication as a co-author.
- In [country name] - one must have two or three publications in [a] journal with impact, so you do not say what or why; simply you must do it. You do not solve the problem [regarding] predatory journal[s]
- Predatory journals are a disruption of an honorable initiative that should have been primarily created by major publishers and respected universities. The power behind owning the rights of scientific discoveries became untamable in the increasingly faster internet era. The results are predatory journals, Sci-Hub, irregular papers sharing and lack of interest in traditional publishing by young investigators.

- They invited me to submit a paper. I had just a paper that was rejected by another journal. The publication fee appeared to be fair. Review and handling of the paper were OK. It is now on-line and well received in ResearchGate, so what?
- Because of the monopoly of editor selection, we need the new international journals
- Actually I am not an editor in this said journal. I don't know how my name is listed as one. Will be happy to do whatever from my end if needed or possible to change the journals publication process
- Main reason for publishing this paper is that the Bioelectric Theory is not yet accepted. Therefore, the top journals like Nature having rejected the submission to them of the original Bioelectric Law paper meant that I needed to reach the scientific community with a paper showing it applied to many living cells
- Do you think that the journal I have submitted for is a predatory journal? If yes do you know whether it is possible to withdraw from that journal and submit elsewhere?
- I guess that, even with predatory journals, we still have more visibility and ease in publishing our surveys. More famous magazines are more difficult and do not always accept our articles because they justify that our studies are sometimes more regional, sometimes without the necessary scientific rigor or with several limitations, among others.
- I think if an article reviewed and revised by qualified journal team, it will increase scientific [merit] of the article.
- This [is a] nice topic to help researchers to be aware from publishing work with online journals How do I know if journal is predatory from fees charged?
- I do not know if it is a predatory journal because it has not imposed a payment but only a request for a free offer
- Really it is difficult to know that such [journal] is predatory. This is why I would suggest that the list of predatory journals be published regularly so that scientists [don’t] fall into the trap. The paper you mentioned, it is expensive for a publication and if I knew it well before I was not going to do it because the financing is problematic and we work with insufficient means. We did it to participate in the advancement of science. It was a sacrifice for me. So to the question of whether it is a predatory journal or not because they have asked dearly, it is a difficult question. But the article is read everywhere and I receive message[s] from everywhere as you have discovered me too.
- As it was my first career publication, had to struggle finding the journals at last this journal seemed affordable and a bench mark to start my research careers, really satisfied me.
- After providing article processing charge, the publishing time is so much lengthy.
- I don't accept that Beall's list is evidence based, it is just an opinion.
- So the opinion of one librarian does not make this list legitimate, it only shows that there was some work accomplished compiling it. Not everyone in the science field was asked opinions on what makes a predatory journal so I take Beall's list accordingly. I do feel like there are predatory journals and publishers out to make dollars while compromising the peer review process. I do feel like this journal was sub-par in the peer review process but there were legitimate comments provided from 2 reviewers. I do also believe this journal was clearly just trying to publish materials to generate business. I did realize these shortcomings going into the process
- There is a such thing as paying a fee and then getting published which is considered predatory. However, there is also a growing movement of traditional academic journals and database companies participating in PR smear campaigns against open-access journals and labeling them all predatory because they are threatened by the open-access model. This personally disgusts me because peer-review is very subjective as I have often had articles reviewed and received a score in the 90s by one reviewer and 50s by another reviewer for the same article in the same journal. Good luck with your study.
- I haven't heard the phrase 'predatory journal' but I have come across some that I think meet this definition. I've gotten emails from publishers of random biology topics, usually addressed 'dear researcher,' stating that I am being invited to submit an article. They tend to ask for thousands of dollars for submission so I have never considered submitting to them.
- The conclusions and recommendations of my article are controversial and are not in line with the 'party line.' It was summarily rejected by 2 mainstream journals, without any peer review, based not on merit, but on what I believe to be a political basis. I finally decided it would be best to just pay $300 and get it published somewhere.
- This Journal was not on the list of predatory journals. It however, might not have been a good choice due to limited visibility

- Likely this is a predatory journal. I did look at their editorial staff before I accepted and there was an international cohort of editors, which in retrospect should have been a red flag. I actually thought we weren't going to be charged any money when we wrote the paper since they asked me to write a review article, but by the time they asked for money, we had already put in a lot of effort, so I paid. I am guessing this article will never be found or cited. It will be one line on my CV and that of my medical student, so hopefully it will help him. I am in private practice, so I am sure it won't do anything for me
- I normally strictly avoid publication of original research in any journal that charges fee, particularly so if it is a less known/start up journal that may turn out to be predatory in nature.
- I did not know the issues on the predatory journal at all. The reviewing process was so rapid. The comments from a reviewer were good and reasonable. The processing charge was reasonable. It was rapidly published as open access journal. Many journals require so long to time for the reviewing and publishing processes. Thus, I liked the particular journal. At that time, I did not feel curious. Thus, I submitted one more original article and accepted after the reasonable revision. The reviewing process was not so curious. It is a pity that this journal is a candidate of predatory journal. I was really shocked.
- The journal changed the name
- I have never known about such Journals, I was so suspicious on the way they treated me about publishing. I really did know. However, after I published this journal [was] blacklisted by the university. I am so sorry for myself and the time that I spent to write this and I am happy at this stage in my early carrier I [learned] this. I have more Papers and hopefully I will publish them with very open eyes and I will check before
- This Journal provided significant editorial services (4 rounds) and the manuscript was peer-reviewed. While it is not indexed on PubMed, folks can find it using search engines such as Google
- This list not only protect writers from Predatory journals but also new journals can improve themselves to come [off] from this list
- Being an editor is a good learning experience for me
- I have never been associated with [ommitted] Journal.
- Now this journal [is] getting good and hence the acceptance of article with this journal is getting tough
- I am not on the Editorial Board of this journal.
- I do not believe that it is a Predatory journal. As far I know, main objective of this journal is not only to gain the publication fee.
- [This] is not a Predatory Journal. Articles submitted are subject to strict peer-reviewing. All research articles are reviewed by at least two suitably qualified experts. So please don't consider this popular journal as a Predatory Journal.
- The Beall's list is controversial and many reputed journals have been placed in the list. It has no authenticity. So please continue with your good work
- I don't remember getting anything to review for this journal. If I am listed as an editor I have had no editorial responsibilities.
- I am satisfied with the product. The article is available online at a reasonable cost
- The editor gave me full fee waiver from any kind of charges for publication of the article
- The open access journal is requires the author to pay. It is difficult for those who are not having external funding.
- This journal is not predatory journal. I think that Mr. Beall is wrong in some cases, but I must tell you that I think Mr. Beall is right in some cases.
- When the article was in press I told them to remove my phone number from publication but they did not, also after publication they did not respond to my emails.
- I don't believe [] predatory journals exist.
- They had offered me free publication therefore I had accepted to publish. I am retired with no money to pay

Selected Unsolicited Emailed Comments *

- I totally don't know anything about predatory journals until last week when I attended a research workshop organized by nursing and midwifery council of Nigeria. As a novice, I was just curious to publish my research project, I paid $150 dollars to publish that work, self-funded. But now I am enlightened, I will not fall victim next time. I was enticed by their internet advertisement. I promise to enlighten more people in my place of work to avoid predatory journals.
- I am no longer an editor for this journal. I resigned about a year ago, and asked many times to remove my information from the journal’s website.

- I have resigned the Editor position about a year ago, and have requested many times to remove my name and picture from the journal's website, without hearing anything from the publisher. So I don't know much about this journal. The editorial office usually assign the reviewers. I heard many complaints.
- Being an editor of an international journal for the past 30 years and published around 350 articles in [omitted] I am highly surprised that this bogus person (Mr. Beall) isn't sued in U.S. Federal Trade Commission (FTC) by any American professor or any US Institutions, which alleged deceptive practices.
- I did not bother to notice that they charge a publication fee. I only found out after they sent me the reviews and I made the changes. They then told me they were satisfied and would publish the paper for $1800! At that point I felt stupid, not knowing what was going on upfront. After thinking about it for a week, I decided that it was worth $300 just to put the project to bed. So, I made them a counter offer of $300, and they accepted. So the pay-to-publish is negotiable, if you had not already heard that. Thanks again for investigating this issue.
- Interesting to me to be selected for this. I'm actually former [omitted] medical faculty and at one time gave grand rounds @ UCSF. Truly, placing this study in this journal was a great relief for me by way of getting around the prejudicial idiocy and rigidity of several mainstream periodicals. Indeed, had they even been willing to review it, I was would have suffered asinine reviews. No question that there is a problem in the on-line era of open access publishing. Plenty of junk science from largely [omitted] funded sources, but in some cases, the open access venue is a useful end run option (rather like using Amazon v. a retail [shop] only to find the inventory insufficient). I pressed for and received good edits and then got a tidy PDF I've used to pave the way for numerous grand rounds appearances in med schools.
- Found this interesting. Do you know for sure that the journal I have submitted for is a predatory journal? If yes do you know whether it is possible to withdraw from that journal and submit elsewhere?
- I have searched for the meaning of predatory journals and learned that [omitted] has been accused of being predatory. I then went through my list of journals and those who have published my work and could not find OMICS although I recognise the name. By searching in my files, I found [omitted] in a dead box with my comments in capitals to not deal with them. Something must have steered me away from them. Academics who pay to be published are buying promotion because it is the only thing they can do to help themselves. Their talent is measured by the number of publications. On line publishing has arisen to serve that market. As well as asking about on-line journals, it would be interesting to question academic institutions on their means of evaluating researchers and teachers. I sense sour grapes amongst the old fogeys who do not have a business model to compete with innovation. [Omitted] has clearly annoyed me in the past and they are run from India, as are most on-line journals, but I understand their ambitions. I have a factory in India and its value is the very high quality of brain power and honesty which I do not find in Europe or the USA. I am curious to learn what you will discover.
- Your research is very interesting and I would like to be familiar with the final results. I hope that is not be problem for you. I must tell you this: I was contacted many times from different journals and asked to write a paper. E-mails comes from unknown addresses and are not signed by anyone. For me, this is real examples of predatory journal.
- I wanted to provide some additional perspective on why I chose a "pay to publish" journal. First, I never heard of Bealls List. […omitted] I certainly never felt preyed upon during my interaction with the journal. When I saw the online version of the article I was impressed with the quality. Second, I am not a novice to scientific authorship so I know the ropes. I submitted my manuscript to 2 "mainstream" journals and was summarily rejected, without any peer review, by what sounded like the managing editor. The reasons given were clearly because my criticisms and conclusions did not agree with "party line." I won't bore you with those details. I am no longer in academia and undertook this research project solely to answer a question that came up in my clinical practice. I succeeded in answering that question, and I thought the information was worth distributing. I paid $300 to publish, and consider the money well-spent, although I am not sure anyone will ever read it. But it gave me closure to a process that had left a bad taste in my psyche. Sorry to bore you, but thanks for letting me vent. PS: Good look getting the survey published. I would love to read the paper when you finish the study.
- I am not aware I am listed on the editorial board of this journal. I may have edited a paper that was published in that journal, but I do not recall it. I am concerned about predatory journals and would like to cooperate with you. Often, I review articles for editing companies when the authors are not identified
- Our mission is to publish works of researchers from third world countries without charging article processing fees. We are having some challenges with financial sustainability and our organization is

currently seeking a grant of at least $2 million. We want a complete waiver policy that will ensure that authors from low-income countries will be able to publish without stress.

- We are yet to publish a single article this year. In view of this kind of situation, my main perspective as an editor of medical journals and open access publishing generally is that researchers from low-income countries are not able to participate fully in the international research community. To be sure, we have received over four hundred and seventy-five articles for publication in our International Journal of Public Health and Epidemiology since the beginning of 2018, but authors are complaining of their inability to pay even the smallest article processing fees. We have tried to make them understand that it costs money to produce a peer-reviewed, edited, and formatted article that is ready for online publication, and to host it on a server that is freely accessible without barriers around the clock. We also reduced our article processing fee as much as possible, yet these researchers want us to publish their articles free of charge. Well, it is not their fault. These people are really having financial problems!
- My perspective as an editor of medical journals/publishing is that it is difficult to sustain especially when you focus on the developing world. We have written to a number of organizations explaining our mission, but none has responded in the positive. We really want to break the barriers that hamper researchers and scientists from publishing their works but we are hampered ourselves. We need help in order to achieve our dream of providing free publication to authors based in any of the following countries which were classified by the World Bank as Low-income economies or Lower-middle-income economies.

* Additional 19 comments received which thanked us for the invitation to participate or let us know the individual completed the survey
